# Mammalian pathogenicity and transmissibility of low pathogenic avian influenza H7N1 and H7N3 viruses isolated from North America in 2018

**DOI:** 10.1080/22221751.2020.1764396

**Published:** 2020-05-24

**Authors:** Jessica A. Belser, Xiangjie Sun, Nicole Brock, Joanna A. Pulit-Penaloza, Joyce Jones, Natosha Zanders, C. Todd Davis, Terrence M. Tumpey, Taronna R. Maines

**Affiliations:** Influenza Division, National Center for Immunization and Respiratory Diseases, Centers for Disease Control and Prevention, Atlanta, GA, USA

**Keywords:** Ferret, mouse, transmission, influenza, LPAI, avian

## Abstract

Low pathogenic avian influenza (LPAI) H7 subtype viruses are infrequently, but persistently, associated with outbreaks in poultry in North America. These LPAI outbreaks provide opportunities for the virus to develop enhanced virulence and transmissibility in mammals and have previously resulted in both occasional acquisition of a highly pathogenic avian influenza (HPAI) phenotype in birds and sporadic cases of human infection. Two notable LPAI H7 subtype viruses caused outbreaks in 2018 in North America: LPAI H7N1 virus in chickens and turkeys, representing the first confirmed H7N1 infection in poultry farms in the United States, and LPAI H7N3 virus in turkeys, a virus subtype often associated with LPAI-to-HPAI phenotypes. Here, we investigated the replication capacity of representative viruses from these outbreaks in human respiratory tract cells and mammalian pathogenicity and transmissibility in the mouse and ferret models. We found that the LPAI H7 viruses replicated to high titre in human cells, reaching mean peak titres generally comparable to HPAI H7 viruses. Replication was efficient in both mammalian species, causing mild infection, with virus primarily limited to respiratory tract tissues. The H7 viruses demonstrated a capacity to transmit to naïve ferrets in a direct contact setting. These data support the need to perform routine risk assessments of LPAI H7 subtype viruses, even in the absence of confirmed human infection.

## Introduction

Low pathogenic avian influenza (LPAI) viruses of the H7 subtype have been associated with periodic documented outbreaks in gallinaceous birds in North America for decades [1,2]. During this time, LPAI H7 viruses have sporadically crossed the species barrier to cause mammalian infection, including confirmed human infection with H7N2 (United States) and H7N3 (Canada and Mexico) subtype viruses [3]. LPAI H7 virus infection in animals is not typically associated with severe disease; despite this, every LPAI outbreak represents an opportunity for acquisition of molecular determinants of virulence that can lead to the generation of highly pathogenic avian influenza (HPAI) viruses. Outbreaks characterized by LPAI-to-HPAI events in the United States were most recently reported in Indiana (H7N8) in 2016 and Tennessee (H7N9) in 2017 [4,5], highlighting the capacity of LPAI H7 subtype influenza viruses to acquire enhanced virulence [2]. As such, continued monitoring and assessment of LPAI H7 viruses represent a critical component of pandemic preparedness efforts, both to ensure that humans are not exposed to LPAI viruses and that these viruses do not acquire properties that increase their adaptation to mammals.

In 2018, two LPAI H7 subtypes were associated with poultry outbreaks in the United States: H7N1 virus in turkeys and chickens in Missouri and Texas, respectively, and H7N3 in turkeys in California [6]. While LPAI H7N3 viruses have been associated with several independent outbreaks in poultry throughout North America over the past 20 years, the detection of LPAI H7N1 virus in 2018 represents the first reported identification of this virus subtype in commercial poultry flocks in North America. No HPAI viruses were detected during the outbreaks in 2018, and no human infections were reported. However, emergence of HPAI from LPAI H7N3 viruses during outbreaks in Chile, Canada, and Mexico [7–9], with outbreaks in the latter two countries associated with human infection [10,11], underscores the capacity of LPAI H7 viruses to pose a pandemic threat. Furthermore, the identification of selected LPAI H7 viruses with features indicative of enhanced human adaptation (such as a capacity for virus transmissibility in mammalian models, detectable binding to human-like sialic acid receptors, and a shift in threshold pH for fusion compared to viruses from wild birds) [12–16], demonstrates the need to monitor these viruses for adaptation properties and their ability to overcome host barriers and species restrictions [17], even with the absence of confirmed human infection.

Here, we evaluated the mammalian pathogenicity and transmissibility of representative viruses from LPAI H7N1 and H7N3 outbreaks that occurred in North America in 2018. Viruses from both outbreaks replicated efficiently in human respiratory tract cells, productively infected mice and ferrets without the need for host adaptation and exhibited a capacity for virus transmission between ferrets in the presence of direct contact. These findings illustrate the continued pandemic potential of North American H7 subtype influenza viruses.

## Materials and methods

*Viruses.* The North American lineage LPAI H7N1 viruses, A/chicken/Texas/18-007912-2/2018 (ck/TX) and A/turkey/Missouri/18-008108-11/2018 (tky/MO), the LPAI H7N3 virus, A/turkey/California/18-031151-4/2018 (tky/CA), and the LPAI H7N9 virus, A/goose/Nebraska/17096-1/2011 (gs/NE), were propagated in the allantoic cavity of 10-day-old embryonated hens’ eggs at 35°C for 48 h. The North American lineage HPAI H7N3 virus, A/Mexico/InDRE7218/2012 (Mex/7218), and Eurasian lineage HPAI H7N7 viruses, A/Netherlands/219/2003 (NL/219), A/Netherlands/230/2003 (NL/230), and A/Italy/3/2013 (Italy/3), were propagated similarly at 37°C for 26–30 h. For each virus, allantoic fluid was pooled from multiple eggs, clarified by centrifugation, and frozen in single-use aliquots at −70°C. Stocks were serially titrated in eggs to determine a 50% egg infectious dose (EID_50_) by the method of Reed and Muench [18]. All research with LPAI viruses was conducted in a BSL2-enhanced or higher laboratory setting; all research with HPAI viruses were conducted under biosafety level 3 containment, including enhancements required by the Federal Select Agent Program [19].

*Sequence analysis.* Sequence data for tky/MO (EPI_ISL_429249), ck/TX (EPI_ISL_429251), and tky/CA (EPI_ISL_429250) were deposited into the Global Initiative on Sharing All Influenza Data (GISAID) database and alignments of full-length coding sequences were performed using BioEdit software.

*Ethics statement.* All animal procedures were approved by the Institutional Animal Care and Use Committee (IACUC) of the Centers for Disease Control and Prevention and were conducted in an Association for Assessment and Accreditation of Laboratory Animal Care International-accredited facility.

*Cell culture.* The human bronchial epithelial cell line, Calu-3 (ATCC), was cultured and grown to confluence on 24 mm membrane inserts (Transwells; Corning) under submerged conditions as previously described [20]. Cells were inoculated (triplicate wells per virus and condition) apically in serum-free media at a multiplicity of infection (MOI) of 1 or 0.01 based on stock EID_50_ titre for one hour before washing and were then cultured at 37°C or 33°C as indicated. The supernatant was collected at the times specified post-inoculation and frozen at −70°C until serial titration in eggs for detection of infectious virus (limit of detection, 10^1.5^ EID_50_/ml). Statistical significance of viral replication kinetics was assessed using a two-way analysis of variance with a Tukey post-test or unpaired t test correcting for multiple comparisons using the Holm-Sidak method.

*Mouse infection.* Female BALB/c mice (Jackson Laboratories), 6 weeks of age, were anesthetized by vaporized isoflurane and inoculated with 10^6^ (*n *= 11) or 10^3^ EID_50_ (*n *= 6) of virus by the intranasal (i.n.) route in a 50 µl volume. All mice were observed daily for clinical signs of disease. Five mice per group inoculated with 10^6^ EID_50_ of virus were weighed daily for morbidity and mortality assessments; three mice per group inoculated at each virus dose were euthanized at day 3 or 6 p.i. for collection of nose and lung tissues. Eight additional mice per virus were inoculated with 10^6^ EID_50_ of virus by the ocular route in a 5 µl volume [21], and four mice were euthanized at day 3 or 6 p.i. for collection of eye, nose, and lung tissue. All collected tissues were immediately frozen at −70°C until thawing for determination of infectious viral load by serial titration of homogenized tissue in eggs (limit of detection, 10^1.5^ EID_50_/ml).

*Ferret infection.* Male Fitch ferrets (Triple F Farms), 5–10 months of age and serologically negative to currently circulating H1, H3, and B influenza viruses by standard hemagglutination inhibition assay, were used in this study. Ferrets were housed in Duo-Flo Bioclean mobile units (Lab Products) for the duration of each experiment. Ferrets (six per virus) were inoculated by the intranasal route with 10^6^ EID_50_ of each virus in a 1 ml volume and observed daily for clinical signs and symptoms of infection. Nasal washes, conjunctival washes, and rectal swabs were collected from three ferrets on alternate days, and three additional ferrets were euthanized on day 3 p.i. for collection of tissues to evaluate systemic spread of virus, as previously described [22,23]. For assessment of virus transmission in the presence of direct contact, three serologically naïve ferrets were each continuously cohoused with a virus-inoculated ferret starting 24 h p.i., as previously described [24].

*Virus-induced syncytium formation assay.* Vero cell cultures (ATCC) were inoculated with influenza viruses to determine the pH at which syncytia formation occurs, as described previously [25] Briefly, inoculated cell cultures were incubated with 0.1-unit increments of fusion buffer (pH range 5.2–6.0), and were fixed and stained for anti-NP antibody 3 h after fusion induction. The threshold pH for fusion is defined as the highest pH value at which 50% or more syncytia can be achieved.

## Results

*Poultry outbreaks of LPAI H7 viruses in North America in 2018.* LPAI H7N1 viruses have been sporadically detected in North America, primarily from migratory birds and specimens collected during routine surveillance of domestic poultry [26,27]. However, in February 2018, LPAI H7N1 virus was detected in a commercial meat turkey flock in Jasper County, Missouri, and subsequently in a commercial broiler breeder flock in Hopkins County, Texas in March 2018 [6]. Sequence analysis of representative isolates from both states, ck/TX and tky/MO, shared high homology and differed by only three amino acids in the hemagglutinin (HA). Ultimately, over 40,000 birds were depopulated between the two outbreaks.

In contrast to H7N1 subtype viruses, outbreaks of LPAI H7N3 viruses have been reported with increased frequency in North America, with outbreaks in Texas, Missouri, New Jersey, and California over the past 15 years [28]. In September 2018, LPAI H7N3 virus was detected in a commercial meat turkey flock in Stanislaus County, California, leading to the depopulation of over 177,000 birds [6]. A representative virus from this outbreak, A/turkey/California/18-031151-4/2018 (tky/CA), was also found to share high sequence homology with LPAI H7N1 outbreak viruses, differing by 7 amino acids in the HA compared with H7N1 ck/TX virus. Similar to other recently isolated H7 viruses, both LPAI H7N1 and H7N3 viruses lack the 8 amino acid deletion adjacent to the HA receptor-binding site observed among LPAI H7 viruses from 2002 to 2003, and do not possess known markers of mammalian virulence in the polymerase genes.

**Figure 1.**
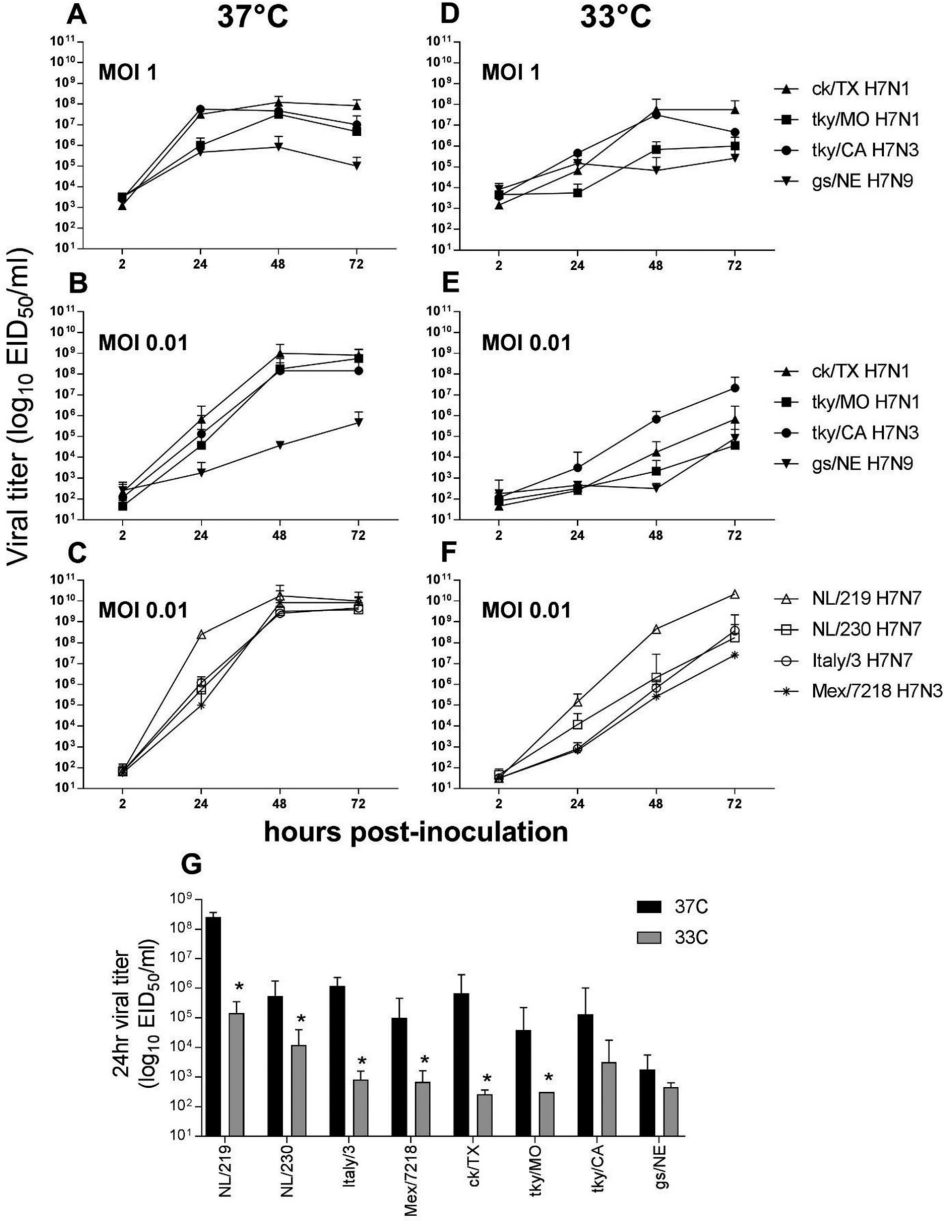
Replication kinetics of LPAI and HPAI H7 influenza viruses in Calu-3 cells. Calu-3 cells were infected apically at an MOI of 0.01 or 1, and cultured at 37°C or 33°C, as indicated with LPAI (A, B, D, E) or HPAI (C, F) viruses. Supernatants were collected at 2, 24, 48, and 72 hrs p.i. and serially titered in eggs for detection of infectious virus. (G), comparison of viral titres 24hrs p.i. from cells infected at a MOI of 0.01 and cultured at either 37°C or 33°C; * denotes *p *< 0.05 between culture temperatures for each virus. The limit of virus detection was 10^1.5^ EID_50_/ml. The mean from triplicate independent cultures per virus plus standard deviation is shown.

*LPAI H7 virus replication in Calu-3 cells.* The human bronchial epithelial cell line, Calu-3, supports replication of a wide range of both human and zoonotic influenza A viruses [20], and has facilitated assessment of the ability of North American and Eurasian-lineage H7 subtype viruses to replicate in a relevant human cell type [20,29,30]. To examine the capacity of LPAI H7 viruses isolated in 2018 to replicate in human respiratory tract cells, Calu-3 cells were infected at a multiplicity of infection (MOI) of 0.01 or 1 with representative H7N1 and H7N3 isolates, as well as a genetically similar 2011 LPAI H7N9 virus (Figure 1(A–B)). All 2018 isolates replicated to significantly higher titres in Calu-3 cells cultured at 37°C compared with the 2011 H7N9 virus at 48 and 72 hrs p.i. at either MOI tested (*p *< 0.01). This difference was most striking at the lower MOI tested (0.01); mean peak titres were 2.5–3.3 logs higher for 2018 H7 viruses than for the 2011 gs/NE virus, while at MOI 1 mean peak titres were 1.5–2.1 logs higher.

In a previous study, an LPAI H7N9 virus associated with a poultry outbreak in 2017 replicated to significantly higher titrs in Calu-3 cells than an HPAI virus from the same outbreak [31], underscoring that virulence in avian species is not uniformly predictive of mammalian pathogenicity or replication capacity in human cells. For comparison, a panel of contemporary North American and Eurasian HPAI H7 viruses associated with both widespread outbreaks in poultry and confirmed human infection were similarly evaluated in Calu-3 cells (Figure 1(C)). HPAI H7 viruses reached mean peak titres of 10^9.5–10.3^ EID_50_/ml in this model, generally higher, albeit only slightly so, than the mean peak titres among all 2018 LPAI H7 viruses examined (>10^8^ EID_50_/ml); differences between LPAI and HPAI viruses were not uniformly statistically significant. These data show that the 2018 LPAI H7 viruses possess a robust capacity for efficient replication in human respiratory tract cells, reaching titres generally comparable to those of HPAI H7 viruses.

Beyond overall viral load, efficient replication at the cooler temperature of the human upper airways represents an additional measure of zoonotic influenza virus adaptation to human hosts [20]. Infection of Calu-3 cells at a high MOI did not reveal overall temperature-sensitive differences (Figure 1(D)). However, the H7 viruses examined here exhibited substantial strain-specific differences with regard to replication capacity at 33°C early (24 h) p.i. following inoculation at the lower 0.01 MOI (Figure 1(E)), likely reflective of both temperature sensitivity and overall magnitude of replicative ability in Calu-3 cells. At 24 h p.i., viral titre differences between 37°C and 33°C among LPAI H7 viruses from 2018 ranged from 1.6 (tky/CA) to 3.4 (ck/TX) logs. This range in variability is comparable to the HPAI H7 viruses examined (Figure 1(F)), with mean titre differences between the two temperatures examined ranging from 1.7 (NL/230) to 3.3 (NL/219) logs at this timepoint. All HPAI viruses replicated to significantly higher titre at 24 h p.i. when cultured at 37°C compared to 33°C, as did the ck/TX and tky/MO LPAI H7N1 viruses (Figure 1(G)). Interestingly, while the tky/CA H7N3 virus reached mean peak titres at 33°C that were within ten-fold of 37°C by 72 h p.i. (a feature generally shared with the Eurasian HPAI H7 viruses tested), the ck/TX and ck/MO viruses were unable to replicate with comparable efficiency at the lower temperature, with mean peak titres 3.1-4.2 logs lower when cultured at 33°C and not 37°C. Collectively, these results indicate that the 2018 LPAI H7 viruses can replicate to high titre in a representative human airway cell type, with replication features similar to HPAI H7 viruses associated with human infection at 37°C. However, strain-specific differences between the LPAI H7N1 and H7N3 viruses were apparent.

**Figure 2.**
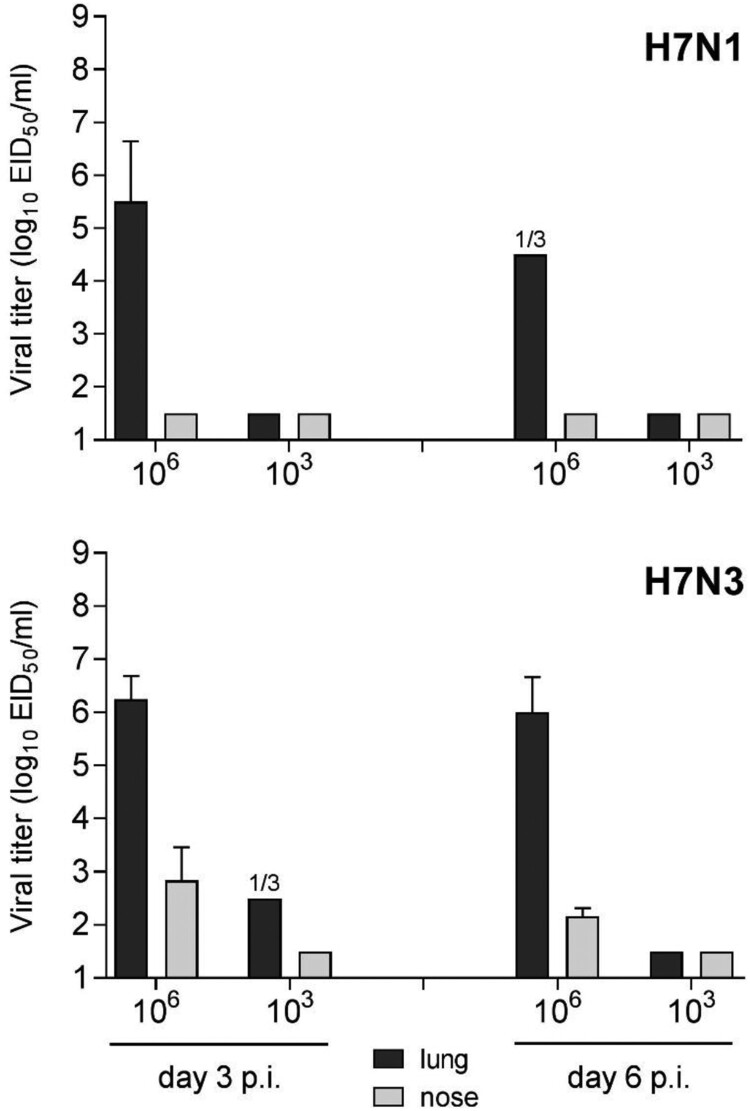
Replication of LPAI H7 viruses from 2018 in mice. BALB/c mice were inoculated with 10^6^ or 10^3^ EID_50_ in 50 µl of ck/TX (H7N1) or tky/CA (H7N3) virus by the intranasal route (*n *= 6 per virus dose) and euthanized on day 3 or 6 p.i. (*n *= 3 per day) for collection of lung (black bars) and nose (grey bars) tissues. Homogenates (*n *= 3 per group) were titered in eggs to measure infectious virus and are reported as mean viral titre plus standard deviation. The limit of virus detection was 10^1.5^ EID_50_/ml. 1/3, one mouse from the group was positive for virus detection.

*Mammalian pathogenicity of LPAI H7 viruses.* North American LPAI H7 viruses typically replicate efficiently in mice without the need for prior host adaptation, and are generally associated with low to moderate morbidity in this species, depending on the inoculating strain [32,33]. To assess the relative virulence of LPAI H7N1 and H7N3 strains isolated in 2018, BALB/c mice were inoculated by the intranasal route with ck/TX or tky/CA viruses, respectively. Mice inoculated with either virus did not exhibit substantial disease, as mean maximum weight loss was <5% for both strains (data not shown). Both viruses replicated to high titres in the lungs of mice following a 10^6^ EID_50_ challenge dose, reaching mean peak titres ≥10^5.5^ EID_50_/ml day 3 p.i. (Figure 2). Unlike the H7N1 virus, the H7N3 virus was detected in the nose on days 3 and 6 p.i. (reaching mean titres of 10^2.8^ and 10^2.1^ EID_50_/ml, respectively), a capacity shared by other LPAI H7N3 North American viruses [29,33]. The absence of robust virus replication at the lower 10^3^ EID_50_ inoculating dose with both viruses is in agreement with other contemporary LPAI H7 North American strains [14,31,34]. Mice inoculated by the ocular route did not become productively infected with either virus, as determined by a lack of detectable infectious virus in eye, nose, and lung tissues days 3 and 6 p.i. (data not shown).

Next, we employed the ferret model, which more closely emulates humans, to examine the capacity of LPAI H7 viruses isolated in 2018 to cause disease. Overall, ferrets inoculated by the intranasal route with either ck/TX or tky/CA viruses exhibited a mild infection. Inoculated ferrets did not exhibit clinical signs such as lethargy, sneezing, or rhinorrhea; transient rises in body temperature did not exceed 1°C above baseline for any ferret. Weight loss was observed in only 1/3 ferrets inoculated with either virus (maximum weight loss of 5.2–5.3%).

**Figure 3.**
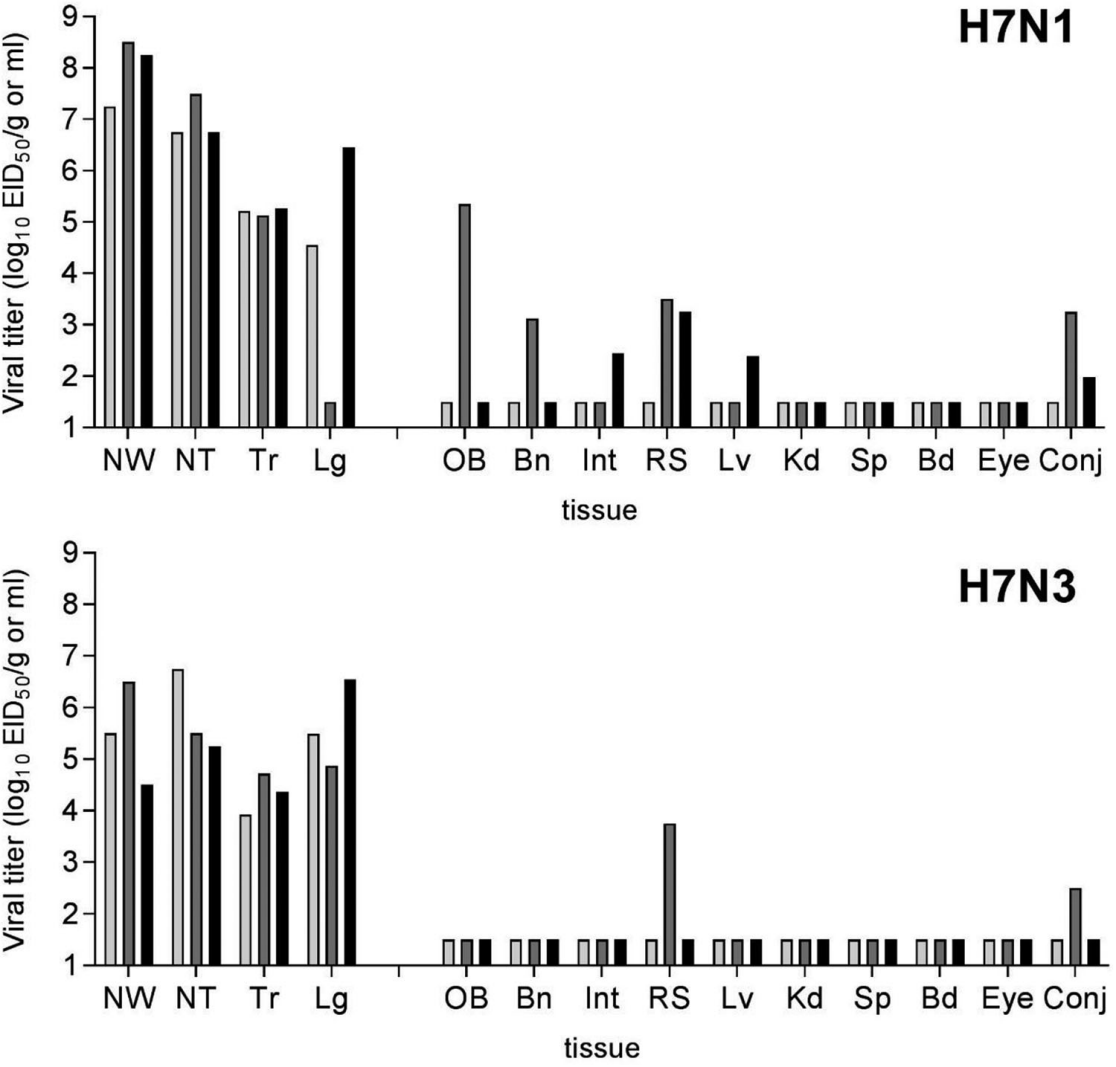
Replication of LPAI H7 viruses from 2018 in ferrets. Three ferrets were inoculated with 10^6^ EID_50_ in 1 ml of ck/TX (H7N1) or tky/CA (H7N3) virus and euthanized day 3 p.i. for collection of systemic tissues. Clarified tissue homogenates were titered in eggs. Bars represent individual ferrets. The limit of virus detection was 10^1.5^ EID_50_/ml. NW, nasal wash; NT, nasal turbinate; Tr, trachea; Lg, lung; OB, olfactory bulb; Bn, pooled anterior and posterior brain; Int, intestine (pooled duodenum, jejunoileum, and descending colon); RS, rectal swab; Lv, liver; Kd, kidney; Sp, spleen; Bd, blood; Eye, pooled right and left eyes; Conj, pooled right and left conjunctival tissue. All tissue titres are expressed per g of tissue with the exception of NW, NT, Bd, Eye, and Conj which are expressed per ml of tissue.

Both LPAI H7 influenza viruses replicated efficiently throughout the ferret respiratory tract, including the lung, in agreement with other North American lineage viruses from this subtype [29,32]. Among respiratory tract tissues with positive H7N1 virus detection on day 3 p.i., mean titres were >10^5^ EID_50_/ml or g (Figure 3). Infectious virus was detected in the olfactory bulb and brain tissue in one ferret possessing the highest viral titres in nasal wash and nasal turbinate samples (>10^8^ and 10^7^ EID_50_/ml, respectively). Infectious virus was also present in pooled intestinal tissue and rectal swabs from 1/3 and 2/3 ferrets, respectively. However, days 1 and 5 p.i. rectal swabs were negative in both H7N1 and H7N3 virus-infected ferrets.

Similarly, mean viral titres in nasal turbinates, soft palate, trachea, and lung were >10^4.3^ EID_50_/ml or g following H7N3 virus infection (Figure 3 and data not shown), supporting a robust capacity to replicate in both the upper and lower respiratory tract of ferrets. Detection of low mean titres (<10^3^ EID_50_/ml) among virus-positive conjunctival samples for both H7N1 and H7N3 viruses, in the absence of virus detection in eye tissue or conjunctival washes collected days 1–5 p.i. (Figure 3 and data not shown), is generally consistent with other LPAI North American H7 viruses [23,35]. Collectively, these data support that LPAI H7 viruses isolated from 2018 can cause mild and transient illness in mammals.

*Mammalian transmissibility and threshold pH for fusion of LPAI H7 viruses.* For over 15 years, North American H7 influenza viruses have demonstrated an ability to transmit between ferrets in a direct contact setting [12,29]; this is inclusive of recent LPAI H7N8 and H7N9 viruses isolated in 2016 and 2017 [31,34]. To examine if LPAI H7 viruses isolated from North America in 2018 have maintained this capacity, ferrets inoculated with ck/TX or tky/CA viruses were cohoused with a serologically naïve ferret starting 24 hrs p.i.; both inoculated and contact ferrets were assessed daily for clinical signs and symptoms of infection, and nasal wash specimens were collected on alternating days.

**Figure 4.**
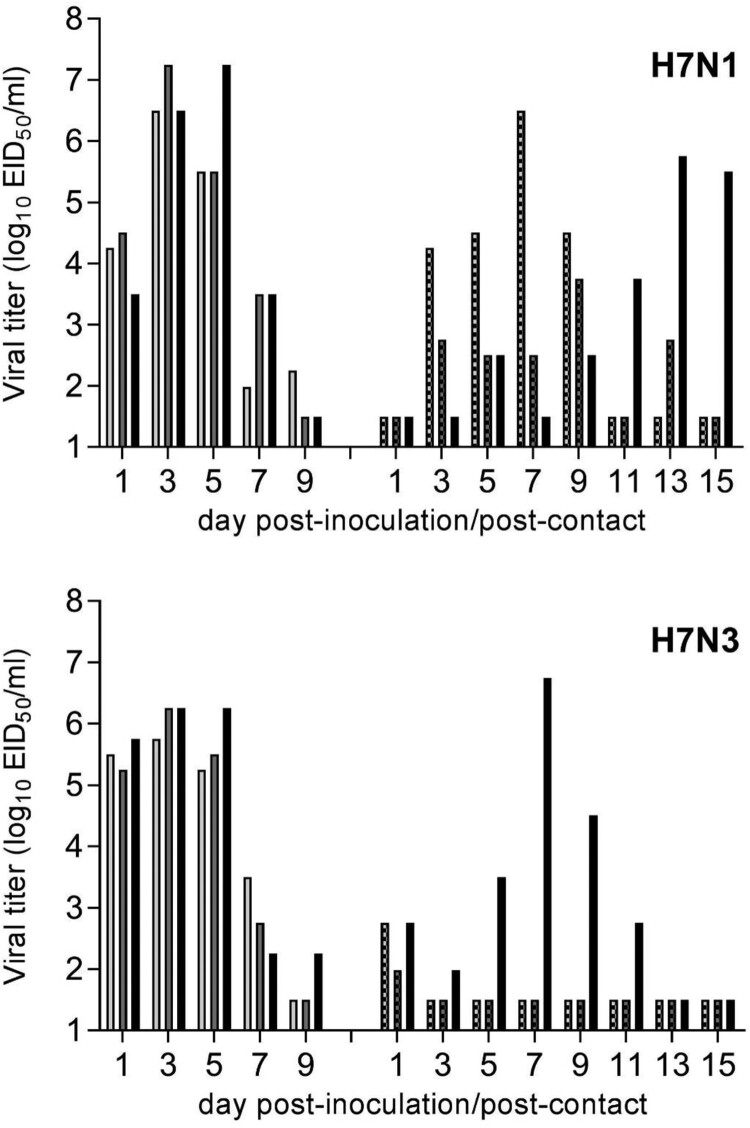
Transmissibility of LPAI H7 viruses from 2018 in ferrets. Three ferrets were inoculated with 10^6^ EID_50_ in 1 ml of ck/TX (H7N1) or tky/CA (H7N3) virus, and nasal washes were collected on alternate days p.i. (left set of bars). Twenty-four hours p.i., a naïve ferret was placed in the same cage as each inoculated ferret and remained in direct contact for the duration of the experiment. Nasal washes were similarly collected (right set of bars). All nasal washes were titered in eggs with a limit of virus detection was 10^1.5^ EID_50_/ml.

The H7N1 ck/TX virus transmitted to 3/3 contact ferrets, with transmission kinetics associated with the timing of peak viral titres detected in nasal wash specimens among inoculated ferrets (Figure 4). All contact ferrets seroconverted to homologous virus by day 21 p.c. In contrast to ck/TX virus, robust virus transmission observed among only 1/3 ferret pairs following inoculation with the H7N3 virus, tky/CA. Seroconversion did not take place in the remaining 2/3 contact ferrets, despite low virus detection (<10^3^ EID_50_/ml) in nasal wash specimens on day 1 p.c., which was likely reflective of virus on the surface of the nose in the absence of productive virus infection. Collectively, these results support that selected LPAI H7 viruses are capable of limited transmission between mammals, despite the absence of confirmed human infection.

Recent studies have indicated a link between influenza virus HA stability and increased adaptation to human hosts [36]; a lower threshold pH for HA fusion is frequently associated with enhanced airborne transmission in mammals [17]. However, the impact of the threshold pH at which HA activation occurs in a direct contact setting is poorly understood. The LPAI H7N1 viruses, ck/TX and tky/MO, displayed pH thresholds of 5.7 and 5.8, respectively, generally comparable to LPAI H7N2 influenza viruses from 2002 to 2003 [14]. In contrast, the LPAI H7N3 tky/CA virus exhibited a lower pH threshold of 5.4, which was similar to an LPAI H7N2 virus associated with an outbreak in shelter cats in 2016–2017 that did not transmit efficiently between cohoused ferrets [14]. These results reveal the heterogeneity present among North American LPAI H7 viruses and highlight the diversity of adaptations that contribute to the host range of zoonotic influenza viruses.

## Discussion

H7 subtype viruses originating from wild bird reservoirs have caused numerous outbreaks in land-based poultry on multiple continents, occasionally resulting in human infection [2]. As risk assessment activities of influenza viruses capable of jumping species barriers include virus, host, and ecological considerations [37,38], it is prudent to employ multi-disciplinary One Health approaches to reduce the incidence and burden of disease in both zoonotic and human hosts [39]. Swift identification of and response to avian influenza outbreaks in poultry prevents the spread of virus to other susceptible animals, reduces the likelihood of human exposure to the virus, and limits the opportunity for virus adaptation in novel hosts [40,41]. Concurrent characterization of avian influenza viruses associated with outbreaks in gallinaceous birds represents a critical component of these activities, to facilitate a better understanding of these emerging viruses to cause disease in and transmit among mammalian hosts. The two documented LPAI H7 outbreaks in the United States in 2018 were caused by an H7N1 virus, not previously associated with outbreaks in poultry on this continent, and an H7N3 virus, a subtype frequently linked with acquisition of molecular determinants of virulence to produce an HPAI phenotype. As such, representative viruses from both outbreaks warranted further investigation in mammalian models.

Selected H7 subtype LPAI viruses from North America have been previously shown to replicate to high titre in human respiratory tract cells and induce innate immune responses early after infection [14,29,30]. However, these studies have largely focused on viruses associated with confirmed human infection. Identification of differential expression of host genes post-infection with LPAI H7 viruses from North America depending on the avian species (chicken, turkey, or duck) from which viruses were isolated [42] underscores a need for a greater understanding of how host adaptation processes can contribute to mammalian pathogenicity should humans be exposed to these viruses. Furthermore, considering the diversity of mechanisms associated with emergence of H7 subtype HPAI viruses from precursor LPAI strains from both North American and Eurasian lineages [2], it can be beneficial to study and contextualize LPAI H7 viruses with genetically similar HPAI strains.

While both LPAI and HPAI H7N1 viruses have caused documented outbreaks in Europe and Africa in recent years [3], H7N1 viruses have not been associated with confirmed human infection and have not been extensively studied for pathogenicity and transmissibility in mammalian models. Two North American LPAI H7N1 viruses isolated during wild bird surveillance efforts were found to cause severe disease in the DBA/2 mouse model [16], a strain with differential susceptibility to influenza viruses compared with the BALB/c model employed here [43]. Eurasian-lineage LPAI H7N1 viruses were found to replicate in mice and were capable of severe disease depending on the virus strain and mouse species employed [44,45]. Additionally, an HPAI H7N1 Eurasian-lineage virus was capable of causing severe disease in ferrets and exhibited limited transmission in a direct contact setting; this virus was capable of supporting an airborne transmissible phenotype following serial passage in ferrets [46]. In contrast to limited information available on North American LPAI H7N1 viruses, the increased frequency of H7N3 viruses detected in North America over the past 15 years has demonstrated that these viruses can replicate efficiently in multiple mammalian models, with selected LPAI and HPAI H7N3 viruses capable of transmission in the presence of direct contact [12,16,29,47].

Both LPAI H7 viruses evaluated here replicated in mice without the need for prior adaptation at a 10^6^ EID_50_ inoculation dose. However, compared with the H7N1 ck/TX virus, the H7N3 tky/CA virus replicated to higher mean peak titres in the lung on day 3 p.i., maintained high viral titres in the lung through day 6 p.i., and was detected in the nose tissue of all tested mice (Figure 2). In contrast, the H7N1 ck/TX virus replicated to higher mean peak titre on day 3 p.i. in the nasal wash and nasal turbinates of ferrets, and was detected at higher frequency in extrapulmonary samples, compared with tky/CA virus (Figure 3). Interestingly, both LPAI H7N1 and H7N3 viruses tested here did not replicate in mice following ocular inoculation in mice and were not consistently detected in ferret conjunctival wash specimens, suggesting that these poultry outbreak isolates had not acquired features associated with the ocular tropism present among numerous H7 subtype viruses [48].

Interpreting the relative pandemic risk of viruses that exhibit limited transmissibility in a direct contact setting is not fully understood [49]. Nonetheless, the continued detection of North American H7 subtype influenza viruses that are capable of transmission in this model suggests heightened mammalian adaptation compared with viruses that do not possess this property. Our observation that both 2018 LPAI H7 viruses evaluated in this study have maintained this capacity further supports this view, though only the H7N1 virus exhibited efficient transmission in a direct contact setting. H7 subtype viruses possessing enhanced transmissibility in ferrets concurrent with relative reductions in murine infectivity and replication have been reported among selected viruses previously [12,32,34], though not uniformly [47], which supports the need for mammalian pathogenicity assessments in multiple relevant species. Furthermore, because a low threshold pH for HA activation has not been shown to enhance virus transmission of multiple North American LPAI H7 subtype viruses [14] in the presence of direct contact, there is a need to better understand how transmission in this setting helps to overcome species barriers facilitating adaptation to mammalian hosts.

Eurasian lineage influenza viruses are responsible for the vast majority of documented human infections with avian influenza viruses. Differences between migratory bird flyways, spatially independent viral evolution between lineages, localization of poultry populations (e.g. live bird markets vs commercial poultry farms), and other epidemiologic considerations likely contribute to this phenomenon [2]. However, recent reassortment of Eurasian and North American lineage viruses, that have led to the generation of HPAI H5Nx viruses associated with poultry infections in North America [50], highlights the role of viruses from both lineages in posing a threat to human health worldwide. Furthermore, recent poultry outbreaks of South American lineage H7 subtype viruses in Chile continue to underscore the genetic diversity of H7 viruses in the Americas [51]. As H7 subtype influenza viruses continue to cause outbreaks throughout North America, ongoing assessment of these viruses represents a critical component of pandemic preparedness efforts.
